# Draft Genome Sequence of a Bioflocculant-Producing Bacterium, Citrobacter freundii IFO 13545

**DOI:** 10.1128/MRA.00524-19

**Published:** 2019-07-18

**Authors:** Priyanka Baranwal, Kazuyuki Kimura, Shanmugam Mayilraj, Seiji Negoro, Masahiro Takeo

**Affiliations:** a Department of Applied Chemistry, Graduate School of Engineering, University of Hyogo, Himeji, Hyogo, Japan; b Hyogo Analysis Center Co., Ltd., Himeji, Hyogo, Japan; c Microbial Type Culture Collection & Gene Bank (MTCC), CSIR-Institute of Microbial Technology, Chandigarh, India; University of Rochester School of Medicine and Dentistry

## Abstract

Here, we report the 5.2-Mb genome sequence of a bioflocculant-producing bacterial strain, Citrobacter freundii IFO 13545, which consists of 5,209,670 bp with a G+C content of 51.5% and 4,853 predicted coding sequences (CDSs). The genes related to the biosynthetic pathway of the bioflocculant were localized on the genome map.

## ANNOUNCEMENT

*Citrobacter* is a genus of Gram-negative coliform bacteria in the Enterobacteriaceae family (1). Currently, the *Citrobacter* genus contains 15 species with 2 recently added members (2–4). Previously, we reported that at least four *Citrobacter* spp. have the metabolic potential to produce a high-molecular-weight chitosan-like polysaccharide during growth on acetate (5). It showed flocculation activity for a kaolin (fine clay powder) suspension (5). Therefore, we are trying to produce it as a bioflocculant. Here, we report the draft genome sequence of one of the bioflocculant-producing strains, Citrobacter freundii IFO 13545, which is available from the Biological Resource Center, National Institute of Technology and Evaluation (NITE; Kisarazu, Japan), as NBRC 13545.

**FIG 1:**
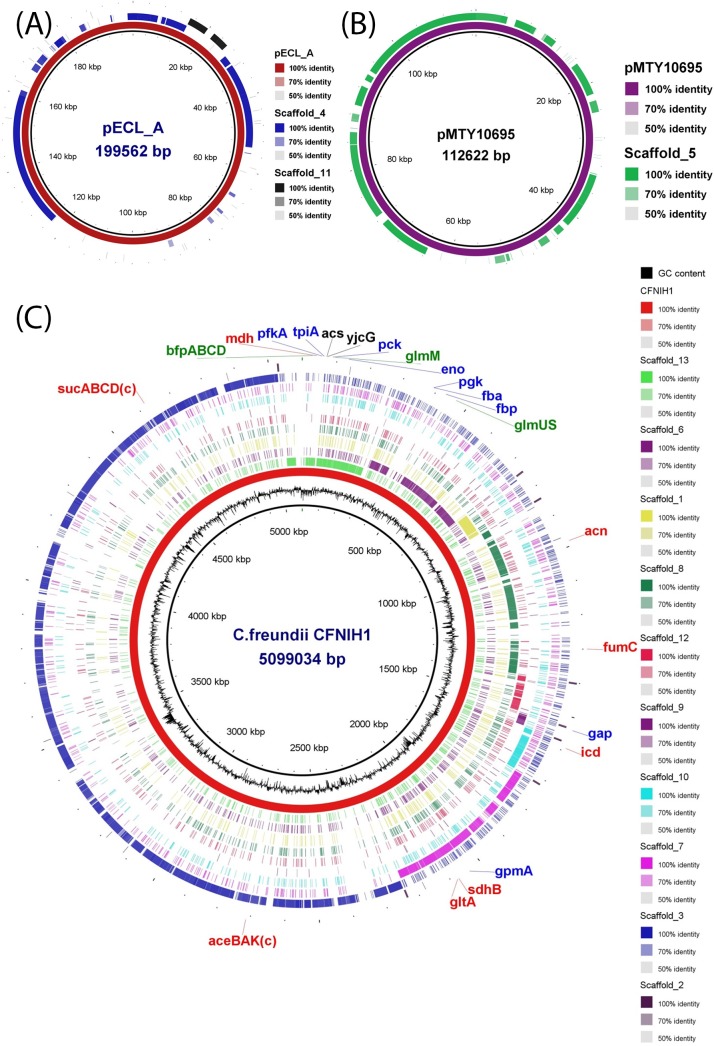
Comparison of the 13 scaffold sequences (Sc1 to Sc13) of C. freundii IFO 13545 with the sequences of plasmids pECL_A and pMTY10695 and the whole-genome sequence of C. freundii CFNIH1 (GenBank accession number CP007557) using the BLAST Ring Image Generator (BRIG) software (15). From the inner circle to the outer circle are plasmid pECL_A (13) and scaffolds Sc4 and Sc11 (A), plasmid pMTY10695 (14) and scaffold Sc5 (B), and G+C content (black), the whole-genome sequence of C. freundii CFNIH1 (red), and scaffolds Sc13, Sc6, Sc1, Sc8, Sc12, Sc9, Sc10, Sc7, Sc3, and Sc2 (C). The putative loci of the biosynthetic pathway genes of the bioflocculant (16) are shown with the gene names (genes encoded in the complementary strand are shown with “(c)” after the gene name). The colors of the gene names (black, red, blue, and green) indicate the genes related to acetate uptake (*yjcG*) and the conversion of acetate to acetyl-coenzyme A (acetyl-CoA) (*acs*), those related to the tricarboxylic acid (TCA) cycle and the glyoxylate cycle (*gltA*, *acn*, *icd*, *sucABCD*, *sdhB*, *fumC*, *mdh*, and *aceBAK*), those related to glycolysis and gluconeogenesis (*pck*, *tpiA*, *pfkA*, *tpiA*, *pgk*, *fbp*, *gpmA*, *gap*, and *fba*), and those related to the hexosamine synthetic pathway (*glmM* and *glmUS*) and the polymerization/secretion (*bfpABCD*) of the bioflocculant, respectively.

The IFO 13545 strain was cultivated at 30°C for 2 days with aerobic shaking in mineral medium, including 10 g liter^−1^ sodium acetate as a carbon source (5), and the total DNA was extracted by repeated freeze-thaw cycles and phenol extraction (6). The draft genome sequence was determined by a combined strategy of shotgun sequencing using a standard run of 454 sequencing technology with a Genome Sequencer FLX system (Roche, Indianapolis, IN, USA) and paired-end sequencing with the Genome Analyzer IIx (Illumina, Inc., San Diego, CA, USA) (7, 8). For the library construction and sequencing reactions, a GS FLX Titanium general DNA library preparation kit and XLR70 sequencing kit (Roche) were used for the FLX sequencer, while a TruSeq DNA kit and a Sequencing by Synthesis (SBS) v5-GA kit (Illumina) were used for the IIx sequencer. The total number of unpaired reads from the FLX sequencer was 403,605 (average, 304 bp), which were *de novo* assembled into 13 scaffolds (*N*_50_ value, 2,649,599 bp) via 121 large contigs (*N*_50_ value, 98,842 bp; contigs with a quality score ≥Q40, 99.6%) using the *de novo* assembler tool (Roche) without any trimming. Then, the ≥3 consecutive identical nucleotide sequences were corrected using the METANI assembler v0.2 (InfoBio, Tokyo, Japan) at 40 positions in total. The total number of paired reads from the IIx sequencer was 35,757,110 (36 bp each), which were used to perform mapping against the 13 scaffolds using MAQ v0.7.1 (9). The final version (named Sc1 to Sc13) totaled 5,209,670 bp. The genome annotation analysis with DFAST (10) showed the presence of 4,853 coding sequences (CDSs), 66 tRNA-coding sequences, and 1 23S-16S rRNA gene cluster (in Sc2) in the sequence. PlasmidFinder (11) and BLAST (12) searches suggested that Sc4 (163,199 bp, 50.3% G+C content) and Sc11 (11,280 bp, 53.7% G+C content) can form one plasmid similar to pECL_A (13) (Fig. 1A), while Sc5 (96,480 bp, 49.8% G+C content) also can form another plasmid similar to pMTY10695 (14) (Fig. 1B). Therefore, the remaining 10 scaffold sequences (4,938,711 bp, 4,580 CDSs, 51.6% G+C content) were compared with the complete genome sequence of C. freundii CFNIH1 using the BLAST Ring Image Generator (BRIG) (15). As shown in Fig. 1C, these scaffolds cover almost all the genome sequence of CFNIH1, and at least 8 copies of Sc2 (the rRNA gene cluster) exist in the scaffold gaps. The complete set of genes related to the biosynthetic pathway of the bioflocculant (16) were found and are shown in Fig. 1C.

### Data availability.

The genome and plasmid sequences of C. freundii IFO 13545, consisting of the 13 scaffolds, have been deposited at DDBJ/ENA/GenBank under the accession numbers BHWY01000001 to BHWY01000013. The BioProject number is PRJDB7528, and the BioSample number is SAMD00143536. The raw sequencing data are available in the DDBJ Sequence Read Archive (DRA) under the accession number DRA008059.
